# Supplementary material for: Allosteric activation or inhibition of PI3Kγ mediated through conformational changes in the p110γ helical domain
Source: eLife. 2023 Jul 7;12:RP88058. doi: 10.7554/eLife.88058 (PMC10392983; doi:10.7554/eLife.88058)
Supplement: Supplementary file 1. [file elife-88058-supp1.docx]

**Supplementary table 1.** Cryo-EM data collection, refinement, and validation statistics **(related to main figure 2)**

|  | p110γ-NB7  EMD- 27627  PDB: 8DP0 |
| --- | --- |
| **Data collection and processing** |  |
| Magnification |  |
| Voltage (kV) | 300 |
| Electron exposure (e/ Å^2^) | 50 |
| Defocus range (nM) | 500-2500 |
| Pixel size (Å) |  |
| Symmetry imposed | C1 |
| Initial particle images (no.) | 795,162 |
| Final particle images (no.) | 149,603 |
| Map resolution (Å) | 3.02 |
| FSC threshold | 0.143 |
| Map resolution range (Å) | 2.6-4.4 |
|  |  |
| **Refinement** |  |
| Initial model used (PDB) | 7MEZ (p110γ only) |
| Model Resolution (Å) | 3.02 |
| FSC threshold | 0.5 |
| Map sharpening B factor | Sharpened locally |
| Model composition |  |
| Non-hydrogen atoms | 8737 |
| Protein residues | 1,066 |
| Ligands | 0 |
| *B*-factors |  |
| Protein | 52.4 |
| Validation |  |
| Mol probability score | 1.29 |
| Clashscore | 5.33 |
| Poor rotamers (%) | 0.0 |
| Ramachandran |  |
| Favored | 98.41 |
| Allowed | 1.59 |
| Outliers | 0.0 |
| R.m.s. deviations |  |
| Bond lengths (Å) | 0.002 |
| Bond angles (°) | 0.490 |
| Model to map fit (CC_mask) | 0.86 |
