# Supplementary material for: Allosteric activation or inhibition of PI3Kγ mediated through conformational changes in the p110γ helical domain
Source: eLife. 2023 Jul 7;12:RP88058. doi: 10.7554/eLife.88058 (PMC10392983; doi:10.7554/eLife.88058)
Supplement: Supplementary file 2. [file elife-88058-supp2.docx]

**Supplementary table 2.** HDX-MS data collection and validation statistics

**(related to main figure 4)**

| Data set | p110γ unphosphorylated | p110γ phosphorylated |
| --- | --- | --- |
| HDX reaction details | %D_2_O=75.5%  pH_(read)_=7.5  Temp=4ºC, 20ºC | %D_2_O=75.5%  pH(read)=7.5  Temp=4ºC, 20ºC |
| HDX time course (seconds) | 3s at 4ºC, 3s, 30s, 300s, 3000s at 20 ºC | 3s at 4ºC, 3s, 30s, 300s, 3000s at 20 ºC |
| HDX controls | N/A | N/A |
| Back-exchange | No correction, deuterium levels are relative | No correction, deuterium levels are relative |
| Number of peptides | 244 | 244 |
| Sequence coverage | 98.4% | 98.4% |
| Average peptide  /redundancy | Length= 15.2  Redundancy= 3.3 | Length= 15.2  Redundancy= 3.3 |
| Replicates | 3 | 3 |
| Repeatability | Average StDev=0.53% | Average StDev=0.57% |
| Significant differences in HDX | >5% and >0.4 Da and unpaired t-test ≤0.01 | >5% and >0.4 Da and unpaired t-test ≤0.01 |
